# Variably protease-sensitive prionopathy in the context of a complex proteinopathy: clinical and neuropathological challenges

**DOI:** 10.3389/fnins.2026.1950078

**Published:** 2026-10-14

**Authors:** Anika Simonovska Serra, Claudia Marina Vargiu, Sigrid Klotz, Evelyn Berger-Sieczkowski, Michaela Kreiter, Marlene Leoni, Alexandra Guttmann-Lattmanig, Lukas Haider, Guenther Regelsberger, Piero Parchi, Ellen Gelpi

**Affiliations:** 1 Division of Neuropathology and Neurochemistry, Department of Neurology, Medical University of Vienna, and Austrian Reference Center for Human Prion Diseases (OERPE), Vienna, Austria; 2 Comprehensive Center for Clinical Neurosciences and Mental Health, Medical University of Vienna, Vienna, Austria; 3 Istituto di Ricovero e Cura a Carattere Scientifico, Istituto delle Scienze Neurologiche di Bologna, University of Bologna, Bologna, Italy; 4 Department of Neurology, Medical University of Vienna, Vienna, Austria; 5 Diagnostic and Research Institute of Pathology, Medical University of Graz, Graz, Austria; 6 Department of Neurology, Medical University of Graz, Graz, Austria; 7 Department of Biomedical Imaging and Image Guided Therapy, Medical University of Vienna, Vienna, Austria; 8 Queen Square Institute of Neurology, University College London, London, United Kingdom; 9 Department of Clinical Neuroscience, Karolinska Institutet, Stockholm, Sweden; 10 Department of Biomedical and Neuromotor Sciences, University of Bologna, Bologna, Italy

**Keywords:** atypical CJD, Creutzfeldt-Jakob disease, FTLD-MND, prion disease, PrP, variably protease-sensitive prionopathy (VPSPr), ladder-like

## Abstract

Variably protease-sensitive prionopathy (VPSPr) is the most recently identified sporadic human prion disease, displaying distinctive clinical and neuropathological features and a unique western blot profile. Clinically, it frequently presents with longer disease duration than classic sporadic Creutzfeldt-Jakob disease (sCJD), neuropathologically with characteristic prion protein (PrP) deposition patterns, and biochemically with variable resistance of PrP*^Sc^* to proteinase K (PK) digestion. Here, we describe clinico-pathological features of two new patients with VPSPr identified at postmortem examination, review current literature, and emphasize diagnostic challenges when combined with other neurodegenerative conditions. As in sCJD, the polymorphism M/V at codon 129 of the *PRNP* gene influences the phenotypic expression, which includes features within the frontotemporal lobar degeneration and motor neuron disease (FTLD-MND) spectrum. Genetic and molecular analysis, as well as postmortem neuropathological examination and western blotting (case 2) were performed. Case 1 with an MM genotype showed a prominent involvement of the motor cortex and striatum, consistent with the pyramidal and extrapyramidal clinical symptoms. The cerebellum was devoid of PrP deposits. Co-pathologies including tau pathology consistent with argyrophilic grain disease (AGD) and limbic-predominant age-related TDP-43 encephalopathy (LATE) were detected as well. Case 2 with a VV genotype displayed a prominent involvement of the cerebellum with microplaques and a slightly different PrP staining pattern, compared to case 1. Both patients showed a prolonged disease duration, 7 years and 3.5 years, respectively. The clinical and neuropathological diagnosis of VPSPr remains challenging, especially in the presence of co-pathologies. The codon 129 M/V polymorphism modulates the clinic and/or pathological phenotypic expression of VPSPr within the FT(L)D-MND spectrum.

## 1 Introduction

Variably protease-sensitive prionopathy (VPSPr) is a rare, likely underdiagnosed human sporadic prion disease with a fatal outcome. First described in 2008 (Gambetti et al., 2008) as a distinct entity among sporadic disease forms, with less than 150 cases reported worldwide to date (Aizpurua et al., 2019; Assar et al., 2015; Baiardi et al., 2022; Cannon et al., 2014; Clemmensen et al., 2024; Erro et al., 2026; Ghoshal et al., 2015; Giaccone et al., 2007; Head et al., 2010, 2013; Huang et al., 2021; Jansen et al., 2010; Kim et al., 2019; Krebs et al., 2007; Notari et al., 2018; Rodríguez-Martínez et al., 2010, 2012; Uchino et al., 2023; Vicente-Pascual et al., 2018; Zou et al., 2010), the clinical diagnosis remains challenging. The common underlying disease mechanism in prion diseases is the conversion of the alpha-helix-rich cellular prion protein (PrP*^c^*) into a pathologic beta-sheet-rich conformer (PrP*^Sc^*) with distinct biochemical and seeding properties, which becomes insoluble and aggregates in the central nervous system (Cobb and Surewicz, 2009). In sporadic Creutzfeldt-Jakob disease (sCJD) PrP*^Sc^* is characterized by its relatively high resistance to proteinase K (PK) and is associated with spongiform change in the gray matter, neuronal loss and gliosis (Baiardi et al., 2019). By contrast, the resistance of PrP*^Sc^* to PK digestion in VPSPr is variable and is significantly lower than in sCJD (Gambetti et al., 2008), resulting in a unique western blot profile (Gambetti et al., 2008). Furthermore, the seeding activity of PrP*^Sc^* in VPSPr is also lower than in sCJD (Diack et al., 2014). This might underlie the negative RT-QuIC results in CSF in most cases. Neuroimaging results (cMRI, FDG-PET) are often inconclusive due to frequent lack of typical cortical and/or basal ganglia hyperintensities, hampering the establishment of the clinical diagnosis (Baiardi et al., 2022; Huang et al., 2021; Kim et al., 2019). FDG-PET may show cortical hypometabolism and thalamic involvement in some cases (Erro et al., 2026), but it is not a specific feature. VPSPr exhibits distinctive histopathological features, and the extent and severity of the neuropathological alterations may depend on the disease duration.

Notably, the disease course differs from sCJD and is considerably longer, with most cases lasting several years. This phenomenon might be explained by the variable resistance of PrP*^Sc^* to proteinase K (PK) digestion and a reduced seeding potency. This, along with slower disease progression, might also explain why examination of 14-3-3 protein in the CSF often remains negative. The clinical symptoms comprise extrapyramidal, psychiatric, cognitive, pyramidal and cerebellar symptoms, and this is strongly influenced by the polymorphism at PRNP codon 129, as occurs in CJD. Based on previously reported cases (Aizpurua et al., 2019; Assar et al., 2015; Baiardi et al., 2022; Cannon et al., 2014; Clemmensen et al., 2024; Erro et al., 2026; Ghoshal et al., 2015; Giaccone et al., 2007; Head et al., 2010, 2013; Huang et al., 2021; Jansen et al., 2010; Kim et al., 2019; Krebs et al., 2007; Notari et al., 2018; Rodríguez-Martínez et al., 2010, 2012; Uchino et al., 2023; Vicente-Pascual et al., 2018; Zou et al., 2010), methionine/methionine (MM) is more frequently associated with extrapyramidal and cerebellar symptoms at onset, while valine/valine (VV) carriers display at onset neurocognitive symptoms mimicking frontotemporal dementia. Heterozygous MV carriers often present with psychiatric and extrapyramidal symptoms (Table 1).

**TABLE 1:** Summary of genotype-phenotype associations in VPSPr according to a literature review^#^.

Genotype	Age at onset (mean ± SD, range)	Disease duration in months (mean, SD, range)*	Sex*	*N*	Symptoms at disease onset	Symptoms during disease progression
MM	68.9 ± 8.9 (55–81)	46.2 ± 21.3 (30–78)	*f* = 56%, *m* = 44%	12***	63% extrapyramidal symptoms, out of these, 83% combined cerebellar and extrapyramidal symptoms	78% pyramidal symptoms suggestive of MND
MV	70.6 ± 11.4 (46–81)	22.5 ± 18.9 (6–72)	*f* = 20%, *m* = 80%**	11	91% psychiatric symptoms, out of these 63% combined psychiatric and extrapyramidal symptoms	55% combined cerebellar and pyramidal symptoms
VV	63.5 ± 8.3 (48–74)	27.8 ± 29.7 (10–120)	*f* = 43%, *m* = 57%	28	86% cognitive and psychiatric symptoms overlapping with FTD features, out of these 13% also extrapyramidal symptoms	64% combined extrapyramidal and cerebellar symptoms; 7% either extrapyramidal or cerebellar symptoms

f, female; FTD, frontotemporal dementia; m, male; SD, standard deviation.

*Sex and disease duration were not available for all cases.

**Missing values from 6 of 11 patients.

***Only 9 patients with reported medical history. # Data have been extracted from references 2 to 21, see reference list.

Here, we present two new cases from Austria: one at an early pathology stage in an elderly woman with a complex proteinopathy and an MM genotype, and the other as a single pathology in a VV carrier. We discuss the diagnostic challenges and review current literature.

## 2 Material and methods

Clinical data were retrospectively retrieved from clinical charts. Postmortem neuropathological examination was performed on formalin-fixed, paraffin-embedded brain tissue sections obtained from multiple brain areas, including all cerebral lobes, basal ganglia, limbic system, brainstem and cerebellum. Tissue blocks were previously decontaminated with 98% formic acid and postprocessed for histological and immunohistochemical analyses. Hematoxylin-eosin and luxol fast blue/nuclear fast red staining as well as immunohistochemistry were performed on selected regions.

### 2.1 Immunohistochemistry

Immunohistochemistry was performed on selected regions applying primary antibodies against different PrP epitopes: anti-PrP 12F10 (mAB, Spibio, A03221, dilution 1:200), anti PrP 6H4 (mAB, Prionics, dilution 1:500) as well as anti-oligomeric α-synuclein 5G4 (mAB, Roboscreen, dilution 1:1000), anti-beta-amyloid (mAB, A3981, clone BAM-10, Sigma Aldrich, dilution 1:600), anti-phospho-tau AT8 (mAB, ser202,Thr205, REF MN1020, Invitrogen, dilution 1:1000), anti-3-repeat tau isoform RD3 (mAB, clone 8E6/C11, Merck Millipore, dilution 1:2000), anti-4-repeat tau isoform RD4 (mAB, clone 1E1/A6, Merck Millipore, 1:800), anti-NF200 (mAB, PA0371, Leica Biosystems, rtu), anti-phosphorylated-TDP43 (pAB, pS309/410, Cosmobio, 1:2500), anti-p62 (mAB, AB_397863, BD Biosciences, dilution 1:1000), anti-CD8 (mAB, PA0138, Leica Biosystems, rtu), anti-CD20 (mAB, PA0200, Leica Biosystems, rtu), anti-CD68 (mAB, PA0273, Leica Biosystems, rtu), and anti-HLA-DR (mAB, ab7856, Abcam, dilution 1:500) antibodies. Diaminobenzidin was used as chromogen.

### 2.2 PrP*^Sc^* purification

Purified PrP*^Sc^* was obtained from about 400 mg of gray matter. Tissues were homogenized (gentleMACS^®^ Octo Dissociator) in 2 ml of 2× TEND (20 mM Tris, pH 8.0, 2 mM EDTA, 265 mM NaCl, 200 mM dithiothreitol) and 2 ml of 20% Sarkosyl, flash-frozen at −80 °C and sonicated, then cleared by ultracentrifugation at 16000 rpm (Beckman rotor SW55Ti) for 25 min at 4 °C in polyallomer tubes. Supernatants (S1) were layered over 1 ml of sucrose solution (20% sucrose in H2O) and ultracentrifuged at 45000 rpm for 143 min at 4 °C. After removal of the supernatant (S2), pellets (P2) were kept overnight at −20 °C in 100 μl of TBS and then resuspended in 0.9 ml of 1× TEND, 10% NaCl, and 1% Sarkosyl through three cycles of freezing and thawing after a 2 min of sonication (40% amplitude). Samples were again laid over a sucrose cushion after adding 1 ml of 1× TEND, then centrifuged in a polycarbonate tube at 45000 rpm for 143 min at 18 °C. After discarding the supernatant (S3), pellets (P3) were resuspended in 200 μl of lysis buffer (100 mM Tris, 100 mM NaCl, 10 mM EDTA, 0,5% Nonidet P-40, 0,5% sodium deoxycholate) at pH 6.9, kept overnight at −20 °C, and then dissolved through two cycles of freezing, thawing, and sonication. Samples were treated for 1 h at 37 °C with proteinase K (PK) (Roche Diagnostics) at a final concentration of 0.0625 U/ml of PK. Digestion was blocked with phenylmethylsulfonyl fluoride (PMSF, final concentration 3.6 mM).

### 2.3 Western blotting

Proteinase K-treated samples were precipitated by adding 8 volumes of methanol and incubating overnight at −80 °C, centrifuged, dissolved in SDS-PAGE sample buffer (final concentration: 3% SDS, 4% β-mercaptoethanol, 10% glycerol, 2 mM EDTA, 62.5 mM Tris) and boiled at 100 °C for 6 min before gel loading. Proteins were resolved in 15% acrylamide gels (Criterion, Bio-Rad) and transferred to Immobilon-P membranes (Millipore Corp., Billerica, MA, USA). After blocking in 10% nonfat milk in Tween-Tris-buffered saline, membranes were incubated overnight with the monoclonal anti-PrP antibody 3F4 (BioLegend, San Diego, CA, USA, working dilution 1:10000), which maps to residues 109–112. After four washing steps in Tween-Tris-buffered saline, membranes were incubated for 1 h with an anti-mouse secondary antibody conjugated to horseradish peroxidase (Cytiva, Marlborough, MA, USA; working dilution 1:4000) and rewashed four times in Tween-Tris-buffered saline. The immunoreactive signal was detected by enhanced chemiluminescence (Immobilon Western Chemiluminescent HRP substrate; Merck Millipore, Milan, Italy) on an Amersham ImageQuant 800 camera (Cytiva, Marlborough, MA, USA).

### 2.4 *PRNP* sequencing

Genomic DNA was isolated from peripheral blood and fresh frozen tissue using QIAamp DNA Mini Kit (Qiagen, Hilden, Germany) according to the manufacturer’s instructions. The coding region (exon 3) of the prion protein gene (*PRNP*) was amplified by polymerase chain reaction (PCR) using the following specific primers:

Sanger:

Forward: 5′-[CAGGAAACAGCTATGACGGGACTCTGACG TTCTCCTCTTC]-3′

Reverse: 5′-[GTAAAACGACGGCCAGTCATGGCACTTCCC AGCATGTAG]-3′

Forward: 5′-[CAGGAAACAGCTATGACGCTGCAGCAGCT GGGGCAGTG]-3′

Reverse: 5′-[GTAAAACGACGGCCAGTGCTGGAAAAAGA TTAGAAAGATGG]-3′

RFLP analysis Codon 129:

Forward: 5′-[GTGGCCACATGGAGTGAC]-3′

Reverse: 5′-[AACGGTGCATGTTTTCAC]-3′

Polymerase chain reaction amplifications were performed in a total volume of 50 μl containing 5 μl genomic DNA, 10 μl Primer-Mix [2.5 pmol/μl], 5 μl 10× polymerase buffer, 5 μl dNTPs [2.5 mmol], 2.5 μl DMSO, 22.25 μl nuclease free water and 0.25 μl Hot Star Taq DNA Polymerase (Qiagen, Hilden, Germany). The amplification protocol consisted of an initial denaturation step at 95 °C for 15 min, followed by a touchdown PCR phase of 10 cycles. This phase started with an annealing temperature of 65°C, which was decreased by 1°C per cycle until a touchdown temperature of 55°C was reached. Subsequently, 30 additional cycles were performed with a constant annealing temperature of 55°C. Each cycle included denaturation at 94 °C for 30 s, annealing for 30 s and elongation at 72 °C for 1 min 30 s. A final extension step was carried out at 72 °C for 6 min. The presence and quality of the PCR products were verified by electrophoresis on an 1% agarose gel stained with GelRed (Biotium, Fremont, CA, USA).

To screen for the M129V polymorphism, restriction fragment length polymorphism (RFLP) analysis was performed. Amplicons were digested with the restriction endonuclease NSPI (New England Biolabs, Ipswich, USA) at 37 °C for 3 h according to the manufacturer’s protocol. The resulting restriction fragments were resolved on an 1% agarose gel to determine the genotype based on the characteristic cleavage patterns: Genotype 1, 129MM (Homozygous); Genotype 2, 129VV (Homozygous); Genotype 3, 129MV (Heterozygous). To confirm the RFLP results and screen the entire coding sequence for rare variants or mutations, bidirectional Sanger sequencing was conducted. Prior to sequencing, PCR products were purified using Qiaquick Gel Extraction Kit (Qiagen, Hilden, Germany) to remove residual primers and dNTPs. Cycle sequencing was performed using the BigDye Terminator v3.1 ready Cycle Sequencing Kit (Applied Biosystems, Waltham, MA, USA) with the following primers:

Forward: 5′-[CAGGAAACAGCTATGAC]-3′

Reverse: 5′-[GTAAAACGACGGCCAGT]-3′

Sequencing products were purified with the DyeEx 2.0 Spin Kit (Qiagen, Hilden, Germany) and subsequently analyzed on an SeqStudio Genetic Analyzer (Applied Biosystems, Waltham, MA, USA). Chromatograms and sequence data were analyzed, aligned, and compared against the *PRNP* reference sequence (GenBank Accession No. [NG_009087.1, NM_000311.5]).

### 2.5 Literature research

Literature research was performed in Embase using Emtree terms “Variably Protease Sensitive Prionopathy” and “Creutzfeldt-Jakob Disease.”

## 3 Results

### 3.1 Clinical summary

Patient 1: An 86-year-old woman was referred to the Department of Neurology in May 2025 for atypical parkinsonism with right-sided bradykinesia. No clinical improvement was achieved after treatment with levodopa/benserazide. The patient reported gait disturbance and frequent falls for 7 years, resulting in fracture of the right humerus in 2019. She also had memory problems and urinary incontinence. The neurological examination revealed a mild rigidity, postural instability, diffuse muscle weakness in upper and lower limbs, accentuated tendon reflexes in the right lower limb, right-sided bradydiadochokinesia, bilateral impaired coordination on the finger-to-nose-test. Moreover, the patient suffered from dysphonia/hypophonia, dysarthria, and a vertical gaze palsy. Numbness of all five fingertips on the right hand had been reported. At admission frontal signs including palmomental reflex, palmar grasp reflex, and snout reflex were absent, and the patient did not show pyramidal signs. A MoCA test confirmed cognitive impairment (MoCA-Score 11/30). The patient had a past medical history of breast cancer for which she underwent surgery, chemotherapy and radiation in 2013. Furthermore, she had coronary artery disease with stent placement, paroxysmal atrial fibrillation treated with anticoagulants, renal anemia, and venous stasis dermatitis.

A nystagmography demonstrated a cerebellar oculomotor dysfunction. CSF analysis performed about 2 months after admission to the neurology department, showed normal 14-3-3 protein, a negative RT-QuIC assay and no antineuronal antibodies. Total tau, phospho-tau, beta amyloid 42 and the beta amyloid 42/40 ratio were within the normal range. A DAT-SPECT showed a slight inhomogeneity of the putamina with asymmetrical hypometabolism, but was not considered abnormal. Brain MRI revealed global brain atrophy, ventricular enlargement suggestive of normal pressure hydrocephalus, and periventricular leukoencephalopathy/microangiopathy (Figure 1). Since lumbar puncture alleviated some of the clinical symptoms, a shunt-implantation was suggested. However, her condition deteriorated acutely, preventing surgery. She developed personality changes, apathy, global aphasia and a left-sided Babinski sign, followed by non-convulsive status epilepticus with myoclonus. A new MRI showed cortical diffusion restriction in the left occipital lobe and left thalamus with no corresponding FLAIR abnormality, suggesting an acute infarct, which was not confirmed in subsequent MRI. Parenteral nutrition was started due to severe dysphagia, but her condition continued to decline. She developed a urinary tract infection and aspiration pneumonia, which led to death despite broad antibiotic and supportive treatment. The total disease duration was roughly 7 years.

**FIGURE 1:**
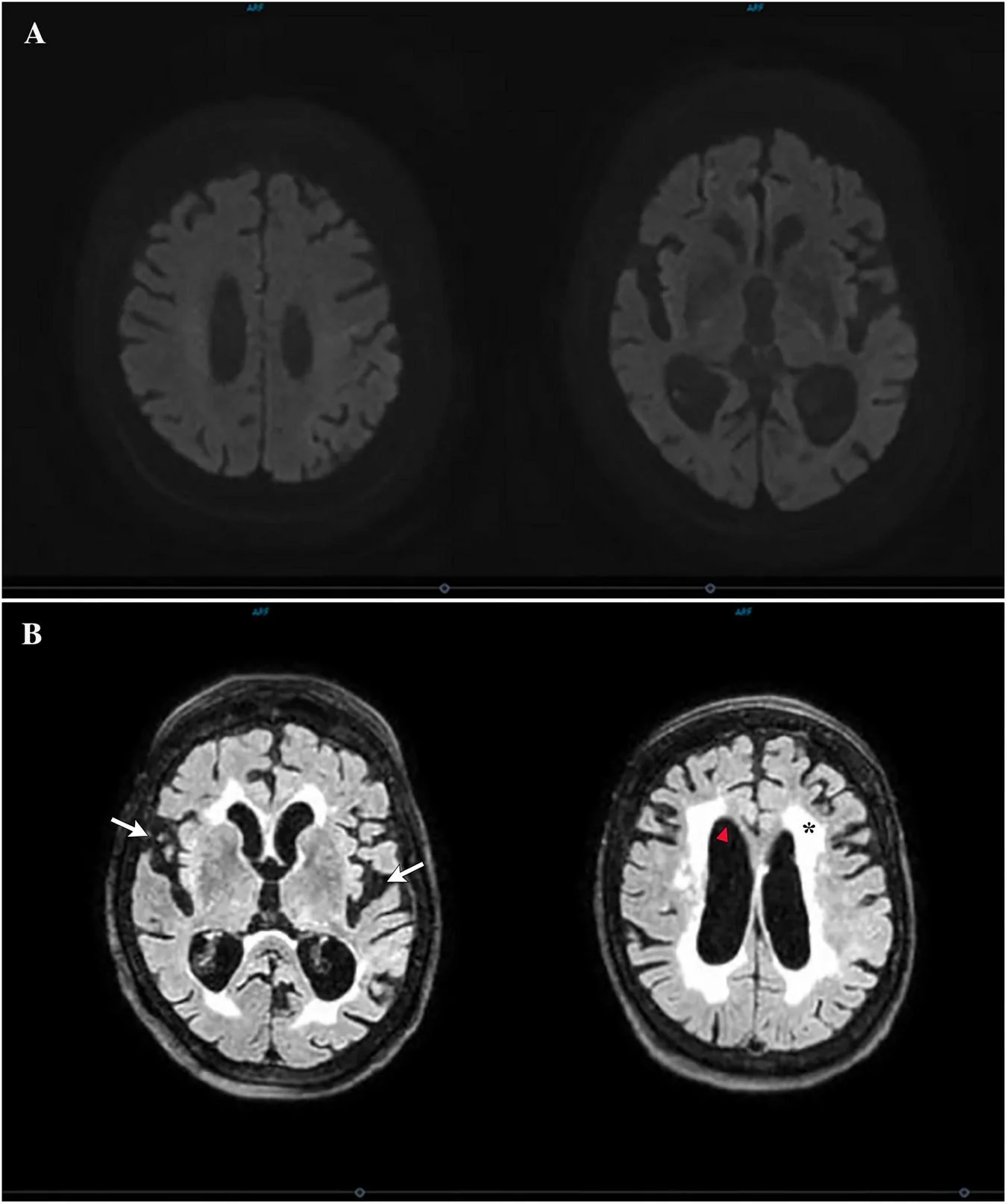
Neuroimaging Brain MRI case 1. **(A)** DWI sequence, axial plane shows no hyperintensities in cortical areas or basal ganglia. There is a moderate ventricular enlargement. **(B)** FLAIR sequence shows periventricular white matter hyperintensities (marked with an asterisk), ventricular enlargement (indicated by a red triangle) and diffuse cortical and subcortical volume loss (marked with arrows). No basal ganglia or cortical hyperintensities are observed.

Patient 2 was a 69-year-old woman who developed subtle progressive cognitive decline since June 2021, which were initially noted by close family members. She was referred to the Department of Neurology in October 2022 because of worsening of the memory impairment in the last 6 months associated with language alteration. This was characterized by prominent word-finding and naming impairment and difficulties following a conversation. In addition, she noticed a gait instability and started walking more slowly to avoid falls. The family members perceived a motor slowing and desorientation as well. Initial neuropsychological evaluation resulted in incipient dementia. Brain MRI revealed ventricular enlargement and no hyperintensities in the cortex or basal ganglia in the DWI or FLAIR. The FDG-PET showed symmetric hypermetabolism with a discrete peri-basal contouring. No triphasic waves were observed on EEG. Analysis of 14-3-3 protein in the CSF was performed twice within 2 months and showed elevated values with 21820 AU/ml and 22510 AU/ml (Ref. < 10000 AU/ml), the RT-QuIC assay was twice negative. Total tau and phospho-tau were slightly elevated. CSF cytology did not reveal any sign of inflammation or tumor cells. No antineuronal antibodies were detected in the CSF or serum. The clinical suspicion of CJD was raised due to subacute memory impairment, language alteration and gait disorder, and increased 14-3-3 protein in CSF, despite the negative RT-QuIC. During the disease, the patient developed tetraplegia and dysphagia. The patient spent 1.5 years in a nursing home and was also supported by family members. The total disease duration was about 3.5 years.

### 3.2 Neuropathological assessment

Case 1:

Unfixed brain weight was 948 g. Gross examination showed a diffuse cerebral atrophy with no focal accentuation and a moderate ventricular enlargement. Bulbar pyramids appeared flattened. Histology revealed a mixture of pathologies. On the one hand, focal and mild spongiform change with small to medium-sized vacuoles was observed in middle and deep cortical layers of fronto-temporal regions, the basal ganglia and the hippocampus. The pre/postcentral region was particularly affected and showed, in addition, prominent neuronal loss, superficial spongiform change and gliosis (Figure 2) as well as degeneration of the descending corticospinal tract. The motor neurons of the brainstem and cervical spinal cord were comparatively well preserved. A mild loss of Purkinje cells was detected in the cerebellum, spongiform change was absent in the molecular layer. Immunohistochemistry for the disease-related PrP^D^ (PrP 12F10) revealed fleecy and freckled deposits on a faint synaptic staining pattern in cortical areas and in the hippocampus. Coarse or granular PrP deposits focally surrounded neurons (Figure 2). Faint deposits were detected in the basal ganglia. In the thalamus focal intraneuronal granular deposits were identified. The cerebellum was devoid of PrP immunoreactivity, even after repeated staining in several cerebellar regions, applying two different antibodies (12F10, 6H4).

**FIGURE 2:**
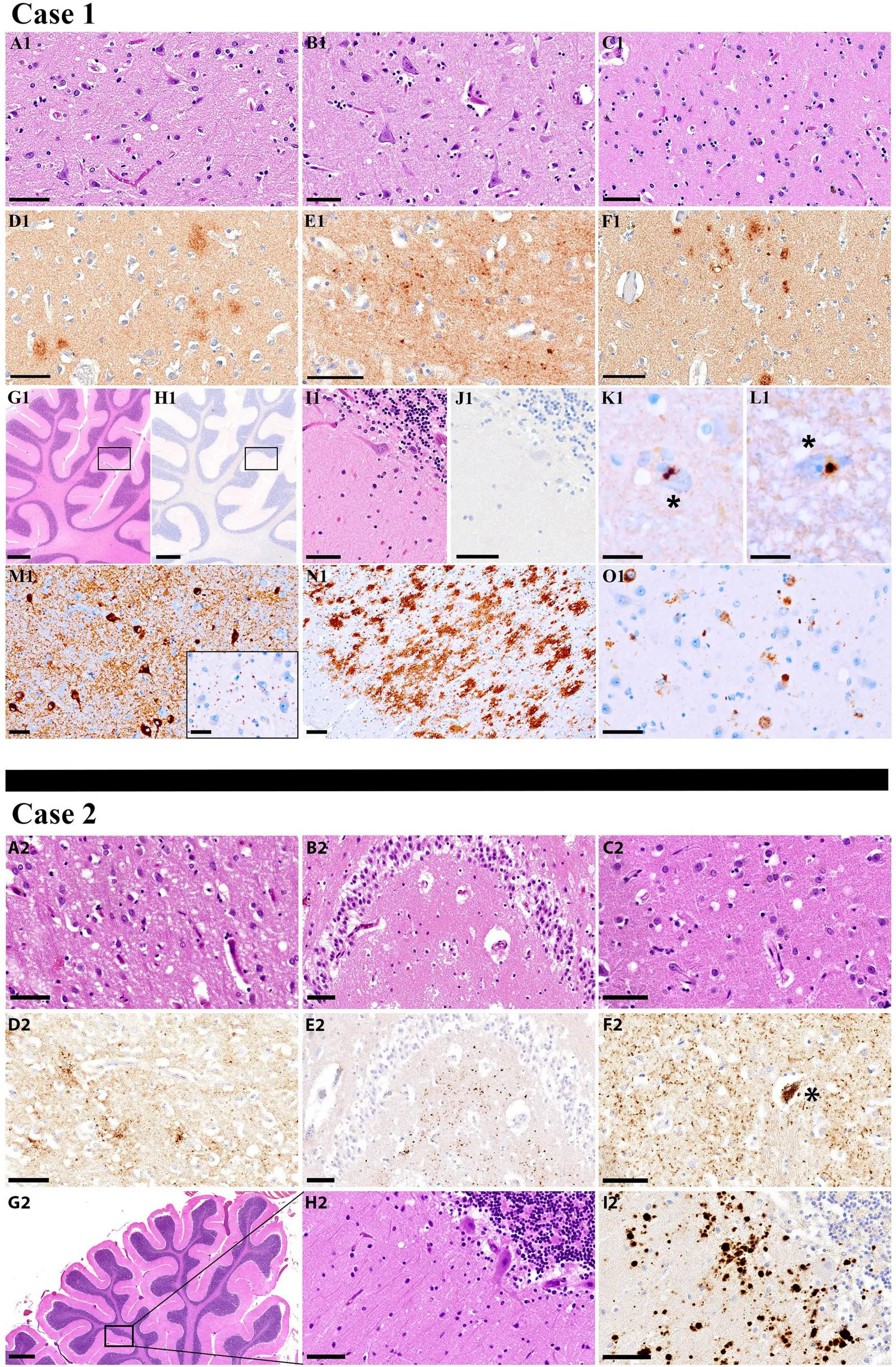
Neuropathology. Case 1 (A1-O1). Mild spongiform change in the frontal cortex (A1), motor cortex (B1) and striatum (C1). Plaque-like and coarse PrP deposits on a faint synaptic background in the frontal cortex (D1), motor cortex (E1) and striatum (F1). Cerebellum devoid of spongiform change in the molecular layer (G1, I1) and without PrP deposits (H1, J1). Intraneuronal coarse deposits in thalamic neurons, indicated by an asterisk (K1, L1). Tau (clone AT-8) staining reveals grains, coiled bodies and ballooned neurons in the amygdala, consistent with argyrophilic grain disease (M1). The inset depicts 4-repeat tau staining of the grains (M1). Beta amyloid immunohistochemistry revealed diffuse and a few coarse senile plaques (N1). Cytoplasmic granular staining of pTDP-43 in the amygdala, consistent with LATE (O1). Case 2 (A2–I2). Edema and mild spongiform change, consisting of small and middle-sized vacuoles in the frontal cortex (A2), in the molecular layer of the dentate gyrus in the hippocampus (B2) and in the striatum (C2). Fleecy and freckled PrP deposits in the frontal cortex (D2), in the molecular layer of the hippocampus (E2) and in the striatum (F2). Intraneuronal PrP deposits in large cholinergic neurons in the putamen are marked with an asterisk (F2). Cerebellum (G2–I2) with spongiform change in the molecular layer (H2) and micro-plaques in the molecular layer (I2). Scale bars: A1–F1, I1–M1, O1 50 μm, inset in M1 25 μm, G1–H1 1 mm, N1 100 μm; A2–F2, H2–I2 50 μm; G2 1 mm.

On the other hand, abundant 4-repeat tau-positive grains were identified in the limbic system on both sides, including the amygdala and the hippocampal CA1 sector, along with ballooned neurons in the amygdala, granular fuzzy astrocytes and oligodendroglial coiled bodies in the temporo-medial white matter, consistent with AGD stage III/III (Saito et al., 2004). Moreover, a mild limbic-predominant age-related TDP-43 encephalopathy (LATE) with few phospho-TDP43 positive inclusions in the amygdala was identified. No phospho-TPD-43 inclusions were detected in the brainstem or in cortical motoneurons. The patient had also mild Alzheimer’s disease neuropathological changes (ADNC; A2, B1, C1) with moderate diffuse plaques and sparse core plaques, mild tau-positive neurofibrillary pathology, and mild amyloid angiopathy involving the frontal leptomeningeal vessels. Diffuse small vessel disease (SVD) with arteriolosclerosis and leukoencephalopathy was identified as well.

In sum, neuropathological changes were consistent with VPSPr with upper motor neuron involvement by morphology associated with prominent tau pathology consistent with AGD, mild LATE and ADNC, and SVD.

Case 2:

Unfixed brain weight was 1250 g. Gross examination showed enlargement of the third and fourth ventricles with no obvious signs of atrophy. Histological examination revealed prominent edema and only mild spongiform change in superficial and deep cortical laminae with middle-sized vacuoles particularly affecting the fronto-temporal regions, which showed moderate neuronal loss and prominent gliosis. The caudate nucleus and the thalamus showed predominant gliosis and less spongiform alteration, which was more obvious in the putamen. In the cerebellum, spongiform change was minimal and focally restricted to the molecular layer.

Immunohistochemistry with anti-PrP antibodies (12F10, 6H4) showed characteristic fleecy and freckled deposits on a faint synaptic background in cortical areas and in the hippocampus. Extracellular and intraneuronal granular deposits were found in large cholinergic neurons of the basal ganglia. In the globus pallidus, axonal immunoreactivity was also identified. In the molecular layer of the cerebellum, characteristic ill-defined coarse deposits, pseudo-plaques and microplaques were observed (Figure 2). No additional protein aggregates were identified; in particular, no alpha-synuclein, phospho-TDP-43 or beta amyloid accumulations, except for delicate tau-positive neurites around spongiform change.

In sum, neuropathological changes were morphologically and immunohistochemically consistent with VPSPr and comparable to the cases reported by Vicente-Pascual et al. (2018) and Erro et al. (2026).

### 3.3 Molecular studies

Western blot analysis was possible only in patient 1. Frozen brain samples from cortical regions showed the presence of PrP^Sc^ with a 19 kDa fragment (type 2) which was virtually devoid of the diglycosylated form, as observed in VPSPr. Two truncated fragments, also suggestive of VPSPr, were also visible, although with a weak signal. The cerebellum was negative, in line with the histopathological findings (Figure 3).

**FIGURE 3:**
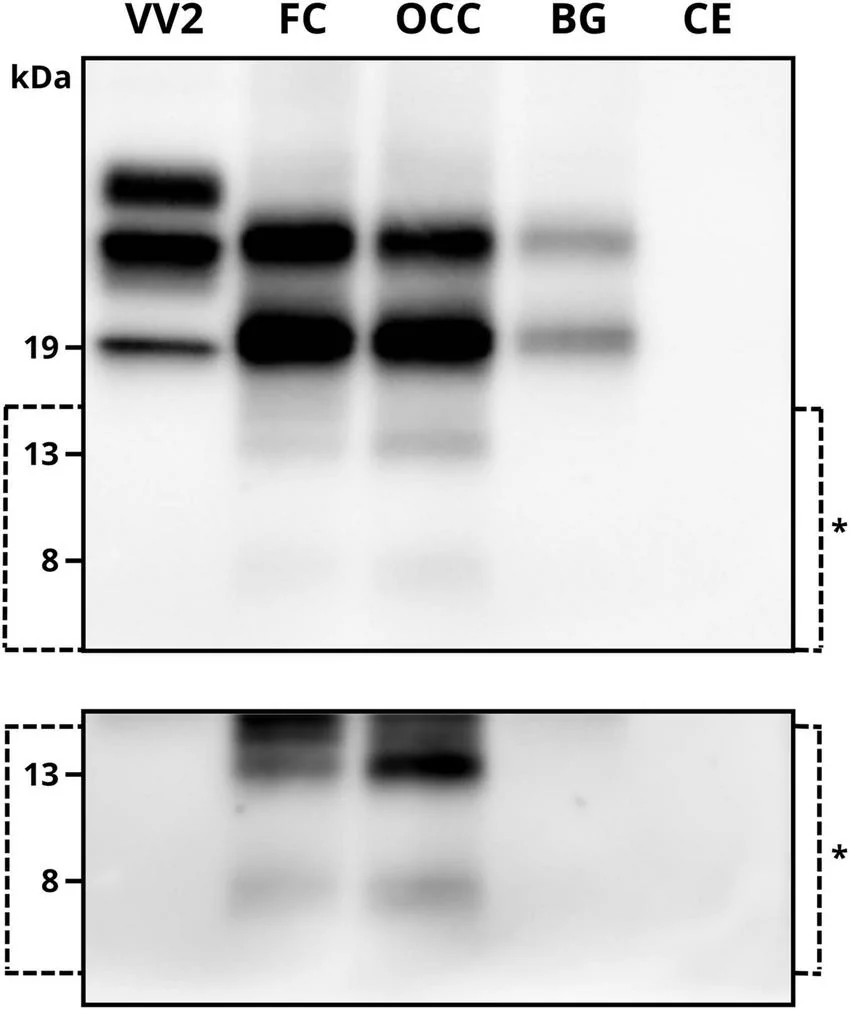
PrP^Sc^ typing by western blot (case 1). As in the sCJDVV2 (lane 1), unglycosylated PrP^Sc^ in case 1 migrates at 19 kDa (PrP^Sc^ type 2). However, in contrast to sCJD, it is virtually devoid of the diglycosylated form. Moreover, two truncated fragments, also suggestive of VPSPr, are visible, although with a much weaker signal (marked with an asterisk). Notably, these two fragments are not seen in sCJDVV2. The immunoblot is probed with the mAb 3F4. The lower box shows the two low-molecular-weight PrP^Sc^ fragments at higher intensity. The two samples from the cerebral cortex show the strongest signal. PrP^Sc^ is present in the basal ganglia in lower amounts than in the cerebral cortex, while the cerebellum is negative, consistent with the histopathological findings. VV2, sCJDVV2; FC, frontal cortex, OCC, occipital cortex, BG, basal ganglia, CE, cerebellum.

### 3.4 Genetic analysis

*PRNP* sequencing showed no mutations in both patients, and the codon 129 polymorphism was MM in patient 1 and VV in patient 2.

## 4 Discussion

Variably protease-sensitive prionopathy is a rare human prion disease which may represent a clinical and neuropathological challenge. Neuropathologically, difficulties may arise from early or topographically restricted alterations, as well as from concomitant neurodegenerative pathologies that may hamper histomorphological and immunohistochemical interpretation.

The cases we present here are two examples of how VPSPr can manifest and the diagnostic challenges it poses. Patient 1 was an elderly MM patient aged 86 years with a complex, non-attributable clinical phenotype (symptoms of normal pressure hydrocephalus and overlapping motor, extrapyramidal and cerebellar symptoms), inconclusive biomarkers (EEG, CSF and MRI) and a challenging neuropathology with restricted changes of VPSPr with a predominant upper motor neuron phenotype by neuropathology sparing the cerebellum, and concomitant tau pathology consistent with AGD, LATE, ADNC, and SVD. Patient 2 presented with rapid progressive dementia and gait disturbances. 14-3-3 protein was positive in the CSF and a prion disease had already been suspected. The disease duration was longer compared to sporadic CJD with VV genotype, and was highly atypical because other ancillary tests, including MRI, EEG and CSF RT-QuIC were inconclusive or negative. Neuropathology showed the characteristic morphological and immunohistochemical features of VPSPr with a dominant fronto-temporal distribution pattern as well as a characteristic cerebellar PrP^D^ deposition pattern. While unfortunately no frozen material was available to confirm the variably protease sensitive properties of the prion protein by western blot analysis in patient 2, morphological and immunohistochemical features were comparable to the WB-confirmed cases described by Vicente-Pascual et al. (2018) and Erro et al. (2026) and consistent with VPSPr.

A detailed histotyping, as performed for other prion diseases, in particular in sporadic CJD, may be also useful in VPSPr. It may help to predict the molecular background at codon 129, particularly when no genetic testing can be performed: a laminar PrP^D^ deposition pattern and intraneuronal granular PrP deposits may suggest the valine genotype, while prominent involvement of the pyramidal system may predict methionine. Previous clinico-pathological reports support these phenotypic associations^25,26^. For example, some MM patients have been reported to present with motor symptoms suggestive of motor neuron disease with neuropathological evidence of motor system involvement (Erro et al., 2026; Vicente-Pascual et al., 2018), such as prominent involvement of motoneurons in the motor cortex, brainstem and spinal cord, along with pyramidal tract degeneration. Those cases had a dominant small plaque-like PrP^D^ deposition pattern (Baiardi et al., 2022). Regarding the MV genotype, a dot-like and, to a lesser extent, a plaque-like PrP deposition patterns has been reported (Baiardi et al., 2022). In patients carrying the VV genotype, PrP mini-plaques and granular deposits arranged in clusters have been described in the cerebellum (Baiardi et al., 2022).

In the present work, both patients had a predominant fronto-temporal distribution of spongiform pathology and predominant thalamic gliosis. The cerebellum was nearly devoid of spongiform alteration in both cases, and the characteristic immunohistochemical PrP^D^ pattern (microplaques, pseudoplaques, coarse deposits) was only detected in patient 2. The cerebellum is particularly helpful for the diagnosis of human prion disorders, for identifying different subtypes, and for histotyping (Parchi et al., 2012). The lack of cerebellar PrP deposits in patient 1 made the VPSPr diagnosis challenging. A lack of micro-plaques in the cerebellum has also been reported previously (Baiardi et al., 2022; Ghoshal et al., 2015; Head et al., 2010). One of the cases was a 93-year-old woman with an MM genotype without clinically evident neurological symptoms (Ghoshal et al., 2015). Another case was a 76-year-old patient with a disease duration of 12 months and an MV genotype, who presented with cognitive decline and developed later motor symptoms (Head et al., 2010). The third case was a patient with an MV genotype, who exhibited cognitive decline, a mood disorder and pyramidal system involvement (Baiardi et al., 2022). The disease duration in the latter case was about 6 months due to an additional infection accompanied by fever (Baiardi et al., 2022). In all these cases, no involvement of the cerebellum was present. However, in other MV cases, a plaque-like PrP^D^ staining pattern has been reported in the cerebellum (Zou et al., 2010).

The lack of cerebellar involvement seems, therefore, to be influenced by the codon 129 polymorphism and the presence of methionine. Previous western blot results indicated that resistance to PK digestion is lowest in VV cases and highest in MM cases, suggesting that differences in the conformation and aggregation of PrP may lead to distinct neurological symptoms and a different PrP staining pattern (Baiardi et al., 2022; Zou et al., 2010).

Moreover, by western blotting, the characteristic band pattern of VPSPr (5 bands around 26, 23, 20, 17 and 7 kDa) can be more clearly recognized using monoclonal anti-PrP antibody clone 1E4 than clone 3F4 (Nemani et al., 2025). This should be taken into account for adequate interpretation. Future studies comparing the pattern in a larger cohort combining different genotypes should be considered.

Another morphological challenge is the characteristic fleecy and freckled PrP-immunohistochemical staining pattern observed in VPSPr, which may mimic the nonspecific co-staining frequently seen in beta amyloid-related senile plaques of AD. However, the PrP microplaques or plaque-like deposits in VPSPr are devoid of classical histological amyloid characteristics, are negative for beta amyloid and do not exhibit a Congo-red positivity or a co-staining with thioflavin S (Baiardi et al., 2022; Kovacs and Budka, 2009).

The western blot pattern of VPSPr with a low molecular weight 7 kDa fragment (Baiardi et al., 2022; Zou et al., 2010) is similar to that observed in Gerstmann-Sträussler-Scheinker disease (GSS), an inherited prion protein amyloidosis, which displays a distinct multicentric PrP-amyloid plaque pathology (Ghetti et al., 1996; Hallinan et al., 2022), and similar to VPSPr, a longer disease duration than CJD and a low transmissibility rate (Nonno et al., 2019). Some authors have therefore postulated that VPSPr represents the sporadic counterpart of GSS.

Another interesting aspect of VPSPr is that for most polymorphic combinations (MM/MV/VV), only mild spongiform change is present in most cases (Baiardi et al., 2022), despite the severity of patients’ neurological symptoms. This apparent discrepancy, which also holds for some other neurodegenerative diseases, may be related to the reduced seeding potency of VPS-PrP. PrP^c^ is a synaptic protein, which plays a major role in synaptic transmission and integration (Kovacs and Budka, 2009). Its misfolding in VPSPr and the production of toxic PrP oligomers may lead to severe and early disruption of synaptic transmission and corresponding motor and cognitive symptoms (Le et al., 2019) beyond the loss of neurons, spongiform change and gliosis observed by microscopic examination (Mallucci, 2009; Singh and Udgaonkar, 2015).

The selective vulnerability of specific systems (Fu et al., 2018), like the pyramidal system in amyotrophic lateral sclerosis (ALS) or the extrapyramidal pathway in Lewy body disease, is also reflected in VPSPr. Vulnerability partially depends on the degree of susceptibility, protein degradation and other signaling and repair mechanisms (Fu et al., 2018). In prion diseases, local vulnerability is underexplored, as various systems are frequently affected simultaneously. Here, the codon 129 polymorphism plays a crucial role in determining phenotypic expression, i.e., clinical and neuropathological distribution of pathology.

An important caveat in the clinical and neuropathological diagnostic work-up is the frequent co-accumulation of other neurodegeneration-related proteins in the elderly and their influence in biomarker interpretation. This was the case in patient 1, where a complex combination of different proteinopathies likely contributed to the neurological symptoms and nearly masked the VPSPr-related changes.

In conclusion, VPSPr is a challenging clinical and neuropathological condition. The influence of codon 129 M/V polymorphism on the phenotypic expression justifies its inclusion in the differential diagnosis of rapidly progressive neurological diseases, including the clinical and/or neuropathological FT(L)D-MND spectrum.

## Data Availability

The original contributions presented in this study are included in this article, further inquiries can be directed to the corresponding author.
